# Mind over Matter: Examining the Role of Cognitive Dissonance and Self-Efficacy in Discontinuous Usage Intentions on Pan-Entertainment Mobile Live Broadcast Platforms

**DOI:** 10.3390/bs13030254

**Published:** 2023-03-13

**Authors:** Shu Zhang, Younghwan Pan

**Affiliations:** Department of Smart Experience Design, Kookmin University, Seoul 02707, Republic of Korea

**Keywords:** discontinuous usage intention, pan-entertainment mobile live broadcast platform, cognition-emotion-behavior intention, self-efficacy, PLS-SEM

## Abstract

The current body of literature indicates a growing trend of discontinuous usage intentions among users of social media platforms. While several factors affecting discontinuous usage intentions have been explored in previous research, the specific factors and mechanisms impacting discontinuous usage intentions among users of pan-entertainment mobile live broadcast platforms remain undefined. This study aims to clarify these factors and mechanisms and to provide both theoretical and practical guidance to users to encourage rational usage of the platform, as well as support the optimization of innovative services offered by the platform’s operator. This study, which is grounded in the theoretical framework of “Cognition-Emotion-Behavior intention,” develops an influencing mechanism model based on cognitive dissonance and self-efficacy. A total of 340 valid samples were collected through questionnaires and analyzed using a structural equation model, which revealed that information overload, service overload, and user addiction had a positive impact on cognitive dissonance, which was moderated by self-efficacy. Cognitive dissonance also had a positive impact on discontinuous usage intentions, again moderated by self-efficacy. These findings expand upon previous research on discontinuous usage intentions among social media users and offer insights into the underlying psychological mechanisms among users of pan-entertainment mobile live broadcast platforms. Additionally, the study provides valuable information for practitioners to consider in the design of the platform, with the ultimate goal of improving user experience and promoting retention.

## 1. Introduction

In recent years, the market scale of the pan-entertainment mobile live broadcast platform has gradually expanded and attracted the attention of academia. According to the 50th Statistical Report on the Internet Development in China released by China Internet Network Information Center (CNNIC), the scale of Chinese live broadcast users had reached 716 million by June 2022 [1]. This shows that the live broadcast market cannot be underestimated. With the upgrading and diversification of user needs, it is insufficient to only rely on the traditional live show and game live broadcast. Therefore, pan-entertainment mobile live broadcast was born, which uses the anchor matrix of star + web celebrity to combine the advantages of the pan-entertainment industry with the characteristics of live broadcast and easily obtain high traffic and user stickiness. With its advantages of high interactivity, strong socialization, and intensive attraction, pan-entertainment mobile live broadcast has become the first choice of many users [2]. As of April 2022, the China pan-entertainment mobile live broadcast industry had reached an impressive active user size of 31.3692 million, according to data from Analysys Qianfan. The industry’s top three platforms, Huajiao Live, Inke Live, and Xiuse Live, have an average quarterly active user size exceeding 10 million each. Huajiao Live emerged as the industry leader in monthly active users throughout 2021, with Inke Live and Xiuse Live following closely behind in popularity [3]. The current pan-entertainment mobile live broadcast industry is in a situation like “even turkeys fly in a hurricane”. On the one hand, a large amount of capital flowed in, and many pan-entertainment mobile live broadcast platforms show a booming development [4]; on the other hand, traditional video sites also entered the field of live broadcast, thus competition among many platforms has become more and more intense [5], from the form of live broadcast to live contents are highly homogenized, leading to the generation of different degrees of aesthetic fatigue of users, and thus the discontinuous usage intention. Discontinuous usage of pan-entertainment mobile live broadcast platforms can be a real problem because it can affect user engagement and retention. Users who do not consistently use the platform are less likely to develop habits or become loyal. This can result in lower user retention rates, reduced user activity, and decreased revenue for the platform. Additionally, discontinuous usage can also impact the content creators on the platform, as they rely on consistent viewership and engagement to build and maintain their audience. If users do not consistently use the platform, content creators may see a decrease in their viewership and revenue potential. Therefore, addressing discontinuous usage is important for both the platform and its users, as it can lead to a more engaging and sustainable ecosystem for all parties involved.

MySpace, once a popular social platform, was acquired due to user churn; Instagram and Facebook, which have seen a decline in user activity in recent years, have incorporated more technical optimization, rewards for participation, and other personalized online service strategies to increase user stickiness [6]. The pan-entertainment mobile live broadcast attracts many users with its novel format, user-produced content, and online real-time interaction, thus generating the social benefits of cultural promotion and image building [2]; however, the limited resources and loss of users due to the users’ discontinuous usage intention affect the long-term effective development of pan-entertainment mobile live broadcast industry [7]. Therefore, to clarify the factors influencing the discontinuous usage intention of pan-entertainment mobile live broadcast platform and its internal logic working is beneficial to guide users to use pan-entertainment mobile live broadcast platform reasonably and reduce its negative effects. At the same time, it can also improve pan-entertainment mobile live broadcast operation services and ensure the healthy and sustainable development of the platform.

From user information reception, entertainment release to cultural dissemination, the scale of pan-entertainment mobile live broadcast platform users and market penetration are gradually expanding [8,9]. In recent years, the rapid development of different types of live broadcast platforms has attracted the attention of scholars, and they explored their dissemination power. Based on the “stimulus-organism-response” model [10], “stressor-strain-outcome” model [11], theory of planned behavior [12], and social comparison theory [13], the previous studies investigated the discontinuous usage intention of social media platform users while fewer studies have been conducted in the field of pan-entertainment mobile live broadcast, and the previous studies have neglected the cognitive psychology and behavioral responses of pan-entertainment mobile live broadcast platform users. From an overview of the current situation of the pan-entertainment mobile live broadcast platform in China, we can find many problems. For example, the threshold for starting a live broadcast on the platforms is relatively low and not limited by the professional ability, location, space, or domain of the anchor; pan-entertainment mobile live broadcast has mixed value shaping, which varies from good to bad, and its live content is highly homogenized; the open comment interaction environment sets up controversial topics and other problems [14]. These can lead to users’ emotional reactions that are inconsistent with perceived expectations. The high return on investment makes pan-entertainment mobile live broadcast favored by the capital market. The platform improves the efficiency of information access through multiple channels, such as multi-clients, big data distribution, and recommendation, but the formation of heterogeneous information will impact users’ existing cognition and create emotional instability and overload psychology [15]. Although discontinuous usage intentions are becoming increasingly prevalent among social media users [16,17], there is still a lack of understanding about the specific factors and mechanisms that contribute to this trend, particularly among users of pan-entertainment mobile live broadcast platforms. While some previous studies have identified several factors associated with discontinuous usage intentions in social media [18,19,20], little attention has been paid to the psychological mechanisms that underlie this behavior among Chinese users of pan-entertainment mobile live broadcast platforms. Given the popularity of these platforms in China, which has the world’s largest mobile internet users [21], it is essential to understand the unique factors that influence discontinuous usage intentions among this user group. The “Cognition-Emotion-Behavior intention” theoretical framework offers a promising approach to understanding the psychological mechanisms driving discontinuous usage intentions. This study aims to apply this framework to the context of Chinese pan-entertainment mobile live broadcast platforms and gain valuable insights into the factors that drive user behavior. By analyzing the influence mechanism of discontinuous usage intention among pan-entertainment mobile live broadcast platform users, the study will propose some recommendations to guide users to use these platforms more rationally and strategies to optimize the operation service of these platforms. This study seeks to answer the following questions:

RQ1: What are the specific antecedents that drive the discontinuous usage intentions of users of pan-entertainment mobile live broadcast platforms in China?

RQ2: To what extent do these factors influence the likelihood of users discontinuing using the platform?

The two research questions seek to identify the factors that lead to users’ intentions to discontinue using a pan-entertainment mobile live broadcast platform in China. They also aim to determine how these factors influence the likelihood of users discontinuing using the platform. These research questions are important because they can help to understand the factors that impact user engagement and retention on pan-entertainment mobile live broadcast platforms, which could be useful for platform operators to improve platforms’ services and retain their users.

## 2. Literature Review

### 2.1. Pan-Entertainment Mobile Live Broadcast Platform

Pan-entertainment mobile live broadcast platforms are online platforms that allow users to access a wide range of entertainment content, such as music, gaming, talk shows, and so on, through live streaming on mobile devices [22]. These platforms typically feature a variety of live streams from different content creators, including both amateurs and professional broadcasters, and allow users to interact with the content and with other users in real time [23]. Some key features of pan-entertainment mobile live broadcast platforms encompass live streaming of a wide range of entertainment content, which includes music, gaming, talk shows, and so forth; a variety of content creators, including both amateur and professional broadcasters; real-time interaction between users and content creators; social features, such as chat rooms and user-generated content; monetization options for content creators, such as virtual gifts and subscriptions; and personalized content recommendations based on user preferences [23].

These platforms have become increasingly popular in recent years, providing users with a convenient and engaging way to access a wide range of entertainment content and interact with other users in real time [24]. With the rise of smartphones and mobile Internet access, pan-entertainment mobile live broadcast platforms have become accessible to a wide range of users and have the potential to revolutionize the way people consume and interact with entertainment content [25]. Prior studies on pan-entertainment mobile live broadcast platforms have focused on various aspects, such as user engagement [26], retention [27], broadcasting intention [28], gifting behavior [29], and comment motivation [30]. The these platforms have an impact on the entertainment industry, and these factors contribute to the success of these platforms [31,32]. While research has focused on the light side factors that influence user engagement and retention, the research on the dark side of discontinuous usage intention of pan-entertainment mobile live broadcast platform users is in a research gap. Therefore, more research is needed on the specific antecedents that drive discontinuous usage intention of users of pan-entertainment mobile live broadcast platforms. Filling these research gaps can help to provide a more comprehensive understanding of the impact of pan-entertainment mobile live broadcast platforms on users and the entertainment industry and can be useful for platform operators to improve their services and retain their users.

### 2.2. Discontinuous Usage Intention

Previous studies mainly centered on the discontinuous usage intention of social media users, and achieved many research results, mainly focusing on the following five aspects. First, the “stimulus-organism-response” model and its application development were used as the theoretical basis for the study of discontinuous usage behavior, and it was suggested that the environmental stimuli (information overload and communication overload) changed the psychological state of the organism and led to discontinuous usage behavioral responses [7], such as controlling the frequency of use, leaving temporarily, and stopping use. Luqman et al. [33] verified the fatigue psychology of Facebook users who stop using it and introduced the effect of technical stress. Second, based on the “stressor-strain-outcome” model, it suggested that the strain variables, such as fatigue and fear of missing out, generated discontinuous usage intentions [11]. Third, in the study of the influence of discontinuous usage behavior in the “cognition-emotion-behavior intention” framework, Dai et al. [34] explored how perceived information overload affects users’ information avoidance intention through the affective factors of fatigue, frustration, and dissatisfaction; Zhou, et al. [35] used data of Weibo users to analyze how information overload and social media fatigue lead to discontinuous usage intentions. Fourth, the behavioral theory was introduced into self-determination theory to integrate the effects of subjective norms, attitudes, and perceived behavioral control on discontinuous usage [17]. Fifth, some researchers have explored how affective factors, such as frustration and dissatisfaction, may impact users’ information avoidance intention, leading to discontinuous usage [36,37].

### 2.3. Cognitive Dissonance Theory

The American social psychologist Festinger [38] proposed the Cognitive Dissonance Theory (CDT), which is based on Gestalt psychology, to analyze the psychological conflict before decision-making and the discomfort afterward. Cognitive and behavioral inconsistencies produce cognitive dissonance and emotional adjustment needs to achieve equilibrium [39]. Cognitive dissonance produces three disordered emotions, including the positive (pleasure), the negative (antipathy, self-accusation), and psychological discomfort (irritability, discomfort), and people usually change their attitudes or behavior to achieve cognitive consistency. Many studies have shown that supportive information contact, information overload [40], contribution overload, differences in system function services [41], and high expectations [42] can produce varying degrees of cognitive dissonance and reduce continuance intention. Cognitive dissonance theory suggests that cognitive consistency is the basic principle of user information processing. Jeong et al. [43] suggested that the cognitive dissonance of users’ opposing views negatively affects discontinuous usage behavior.

### 2.4. Self-Efficacy Theory

Contextual and individual characteristics cause behavioral differences in users’ negative usage, and confidence in self-competence has an important influence on users’ individual behavioral control [44], and the level of self-confidence or Differentiation reaction of positive mental state in coping with obstacles or challenges is known as self-efficacy, whose main intrinsic effect factor is emotion regulation. Self-efficacy regulates motivation, thinking, emotional state, and behavior [45]. Challenging stressors stimulate self-efficacy, whereas hindering stressors reduce individual self-efficacy; high self-efficacy promotes usage intention and low self-efficacy reduces behavioral motivation [46]. The moderating effect of self-efficacy on users’ continuance intention has been demonstrated in financial technology [47], indicating the correlation between mood disorders and continuance intention and the mediating role mechanism [48] of self-efficacy. In this study, we further analyzed the influence of cognitive and affective dimensions on the discontinuous usage intention of the pan-entertainment mobile live broadcast platform users from the perspective of cognitive dissonance and self-efficacy and constructed a model of the influencing factors.

### 2.5. Research Status Analysis

At present, the previous studies mainly focus on factors of the dissemination power of pan-entertainment mobile live broadcast platforms [49,50] while the research on discontinuous usage behavior is carried out by integrating single or several aspects of traditional social media, such as technological stress, environmental stimulation, and emotion. Few studies have integrated the “cognition-emotion-behavior intention” framework and introduced self-efficacy in the theory of planned behavior to further enrich the cognitive and emotional dimensions from the level of the logic of internal factors.

The theoretical framework of cognition-emotion-behavior intention is applicable to this study because it addresses the psychological process of users’ discontinuous usage intention of the pan-entertainment mobile live broadcast platform [51], which is the main focus of this study. The cognitive part of the framework refers to individuals’ thoughts and perceptions about the pan-entertainment mobile live broadcast platform. These perceptions will influence users’ attitudes toward the platform and ultimately their intention to stop using it. The affective part of the framework refers to how individuals feel about the pan-entertainment mobile live broadcast platform. Emotions are influenced by perceptions, which will also affect users’ attitudes and intentions. The behavioral intention part of the framework refers to the likelihood of an individual engaging in a particular behavior, which in this study refers to stopping the use of the pan-entertainment mobile live broadcast platform. By utilizing this theoretical framework, this study will be able to identify the specific factors that influence the behavioral intention to discontinue among users of pan-entertainment mobile live broadcast platforms. This will provide a comprehensive understanding of the psychological process of discontinuing the use of Chinese pan-entertainment mobile live broadcast platform users and help to determine the likelihood of reducing discontinuation. Therefore, this study empirically investigates pan-entertainment mobile live broadcast platform users’ discontinuous usage intention based on perspectives of pan-entertainment mobile live broadcast platform information, platform services, and user addiction, and proposes some advice to guide users to rationally use pan-entertainment mobile live broadcast platform and some strategies to optimize the operation service of pan-entertainment mobile live broadcast platform.

## 3. Research Hypotheses and Model

### 3.1. Research Hypotheses

Information overload refers to the fact that users receive more information than they can process on social platforms. Users of the pan-entertainment mobile live broadcast platform would experience fatigue and loss of control, due to the complexity of the interface, a large amount of similar content, and the diversified profit models that cause the data to be received to exceed their information processing capacity. The pan-entertainment mobile live broadcast platform covers a wide range of content areas, and from the perspective of social networking services, cross-domain information communication increases user stickiness, but increasing users’ pressure of receiving and processing pan-entertainment mobile live broadcast and related information makes users tired. Content domain overload generates the uncontrolled perception of new information exposure and generates cognitive fatigue, anxiety, and boredom [52]. The study [53] found that many Twitter users feel stressed by information overload during social media use. A study found that when users are exposed to a large amount of information, they are more likely to experience cognitive dissonance, which is a feeling of discomfort or tension that arises when individuals hold conflicting beliefs or attitudes [54]. In another study that investigated the impact of information overload on online shoppers’ cognitive dissonance, the study found that information overload led to an increased level of cognitive dissonance among online shoppers, which in turn affected their purchase decisions [55]. Given the above studies, we selected the pan-entertainment mobile live broadcast platform information overload as an affective factor in the environment to study its effect on users’ cognitive dissonance and proposed the following research hypothesis:

**H1:**  *Information overload on pan-entertainment mobile live broadcast platform has a positive influence on users’ cognitive dissonance*.

Service overload occurs when a user is overloaded with too many or too complex services. For example, consumers will feel stressed when they are over-promoted by salespeople during shopping, and Internet users suffer from a lack of patience due to frequent software updates. Based on the characteristics of the pan-entertainment mobile live broadcast platform, service overload is analyzed in two dimensions: services provided by the platform operators are mainly system function overload, i.e., pan-entertainment mobile live broadcast platform or the technical stress generated by software functions exceeding the expected demand, producing tedious information processing fatigue [56]. The process of information sharing on the pan-entertainment mobile live broadcast platform, which users watch, disseminate, and participate in beyond their cognitive and processing abilities [57], thus generating negative emotions [58]. Systemic function overload drives users to spend more time and effort on cognitive enhancement, then users feel functional information acceptance fatigue [59]. A study found that when users are exposed to a large amount of service information, they are more likely to experience cognitive dissonance, which is a feeling of discomfort or tension that arises when individuals hold conflicting beliefs or attitudes [60]. Similarly, a study found that service overload can lead to a higher level of cognitive dissonance among users of social networking sites [61]. The study found that users who were exposed to a large amount of service information on social networking sites were more likely to experience conflicting beliefs or attitudes, which led to cognitive dissonance. The heterogeneous cognitive structure of individuals generates differences in the viewpoint of the social environment of the pan-entertainment mobile live broadcast platform, which lead to cognitive conflict. Based on the above studies, we selected pan-entertainment mobile live broadcast platform service overload as an affective factor in the platform environment to study its impact on users’ cognitive dissonance and proposed the following hypothesis:

**H2:**  *Service overload of pan-entertainment mobile live broadcast platforms has a positive influence on users’ cognitive dissonance*.

Addiction refers to impulse control disorders, namely individuals fail to control their uncontrolled usage behaviors in time and when excessive use causes negative effects on their lives and cannot be controlled in time [62]. A study examined the relationship between smartphone addiction and cognitive dissonance in smartphone use. The study found that individuals who were more addicted to their smartphones experienced a higher level of cognitive dissonance when using their smartphones [63]. Similarly, a study found that online game addiction was positively related to cognitive dissonance among online gamers [64]. There is internal resistance to the continuous usage of the pan-entertainment mobile live broadcast platform, but they cannot stop this usage behavior, thus resulting in cognitive dissonances, such as guilt and discomfort. As the main source of revenue for the pan-entertainment mobile live broadcast platform, advertisements are embedded in many pan-entertainment mobile live broadcast contents, and users will passively receive advertising messages when they access the contents in which they are interested. This may cause users to indulge in the content of the platform and may also cause users to become addicted to the consumption behavior. User addiction can cause negative effects, such as time wasting, social disengagement, mental discomfort, and reduced efficiency in work and study, and cognitive dissonance, such as psychological guilt, comes into being due to the negative effects of addiction on the pan-entertainment mobile live broadcast platform [65]. The following hypothesis was proposed to investigate the impact of user addiction to pan-entertainment mobile live broadcast platform on cognitive dissonance as an individual affective factor:

**H3:**  *User addiction to the pan-entertainment mobile live broadcast platform has a positive influence on users’ cognitive dissonance*.

Cognitive activities involve setting goals and practicing them to achieve expected future values by predicting the possible outcomes of future actions [66]. When users continue to use the pan-entertainment mobile live broadcast platform, they tend to reduce cognitive dissonance by changing their attitudes and behaviors, including behavioral intentions, such as controlling the use frequency, leaving temporarily, and stopping use, and self-motivation relies on goal setting and evaluation and generates effective behavioral interventions, forming a self-efficacy development mechanism [67]. A study by Li et al. (2020) investigated the relationship between cognitive dissonance and continuance intention in the context of mobile instant messaging apps. The study found that cognitive dissonance had a negative influence on continuance intention, suggesting that users who experienced cognitive dissonance were less likely to continue using the app [68]. Another study examined the relationship between cognitive dissonance and customer loyalty in the context of online shopping. The study found that cognitive dissonance had a negative influence on customer loyalty [69]. Therefore, the following hypothesis was proposed by analyzing the influence of cognitive dissonance on users’ discontinuous usage intention of the pan-entertainment mobile live broadcast platform:

**H4:**  *Cognitive dissonance has a positive influence on the discontinuous usage intention of pan-entertainment mobile live broadcast platform users*.

Self-efficacy drives goal construction and effort to achieve goals, and high self-efficacy can promote the cognitive construction of effective actions, and effective behavioral outcomes reinforce self-efficacy [70]. Prior research suggested that individuals correct problems because of cognitive dissonance, and some individuals rationalize them by defending them in the hope of reducing cognitive dissonance without changing their use behavior patterns [71]. Self-efficacy to regulate negative emotions and self-efficacy to feel positive emotions guide cognitive and behavioral intentions through the ability to manage emotional states and beliefs [72]. A study investigated the relationship between self-efficacy and mobile addiction among college students. The study found that self-efficacy had a negative relationship with mobile addiction, suggesting that higher levels of self-efficacy were associated with lower levels of mobile addiction [73]. Another study examined the relationship between self-efficacy and information overload in the context of social media use. The study found that self-efficacy had a negative relationship with information overload, indicating that individuals with higher levels of self-efficacy were less likely to experience information overload [74]. While these studies do not directly investigate the moderating effect of self-efficacy on the relationship between addiction, service overload, information overload, cognitive dissonance, and discontinuous usage intention, they suggest that self-efficacy may play a role in these relationships. Further research is needed to explore the moderating effect of self-efficacy on these relationships in specific contexts. In the face of stimulating environmental stressors, such as pan-entertainment mobile live broadcast platform user addiction, information overload, and service overload, people who are unable to adequately regulate negative emotions may externalize negative emotions [75], such as expressing anger, fear, anxiety, or depression, but positive emotion regulation can mitigate the interference of stimulating environmental factors and cognitive dissonance, and it promotes adaptive behavior and leads to beneficial social interactions and experiences [76]. Self-efficacy indirectly enhances effective action intentions by modulating the relationship between pan-entertainment mobile live broadcast platform user addiction and cognitive dissonance [77], and it positively responds to negative emotions, such as information overload, and drives change in the context, and the higher the influence of positive consciousness, the weaker the sense of cognitive dissonance [78]. In this study, self-efficacy was selected as a moderator of the relationship between emotion and cognition, and the following research hypotheses were proposed:

**H5:**  *Self-efficacy has a moderating effect on the relationship between user addiction and cognitive dissonance among users of the pan-entertainment mobile live broadcast platform*.

**H6:**  *Self-efficacy has a moderating effect on the relationship between service overload and cognitive dissonance among users of the pan-entertainment mobile live broadcast platform*.

**H7:**  *Self-efficacy has a moderating effect on the relationship between information overload and cognitive dissonance among users of the pan-entertainment mobile live broadcast platform*.

**H8:**  *Self-efficacy has a moderating effect on the relationship between cognitive dissonance and the discontinuous usage intention of the pan-entertainment mobile live broadcast platform users*.

### 3.2. Research Model

**Figure 1:**
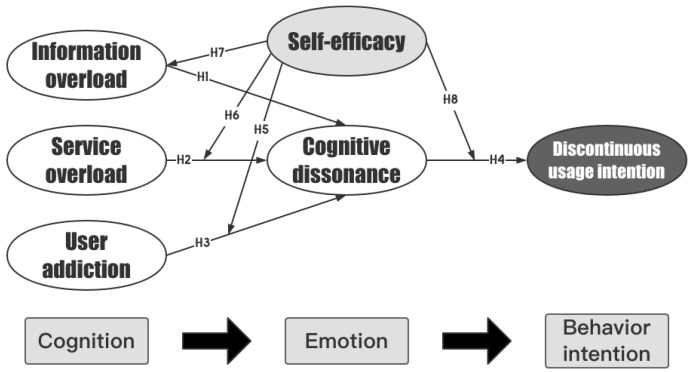
A model of the predictors of the discontinuous usage intention of pan-entertainment mobile live broadcast platforms.

Drawing upon the above hypotheses, this study proposes a research model that encompasses the concepts of cognitive dissonance and self-efficacy to examine the discontinuous usage intention of users of pan-entertainment mobile live broadcast platforms. This model is constructed within the framework of the “cognition-emotion-behavior intention” and is depicted in Figure 1.

## 4. Methods

### 4.1. Questionnaire Design

This study utilized multi-item scales to measure each variable, which offers greater stability and can minimize measurement errors compared to single-item scales. To ensure the scale’s reliability, items for each construct were sourced from existing studies with established reliability and validity. Numerous studies have rigorously tested these scales and demonstrated good reliability and validity. Appropriate textual modifications were made to the original scales to tailor the scales to the context of the pan-entertainment mobile live broadcast platform. The scale of this study uses three to five questions (measurement variables) for the six variables involved in the study hypotheses, with a total of 21 questions. The research questionnaire consists of two parts: the first part is the respondents’ basic personal information, including gender, age, education degree, and frequency of using pan-entertainment mobile live broadcast platform or software. The second part is measurement questions of potential variables in the theoretical model of users’ discontinuous usage intention of short-video social media platforms, using a 5-point Likert scale, in which 1 means “strongly disagree”, 3 means “generally agree”, and 5 means “strongly agree”. The measurement questions of information overload were obtained from Venkatesh [77] and Zhang et al. [79]; service overload from Maier, Laumer, Eckhardt and Weitzel [59], and Maier, et al. [80]; user addiction from Turel [81] and Kwon et al. [82] and cognitive dissonance from Marikyan et al. [83] and Shahin Sharifi and Rahim Esfidani [84]; self-efficacy from Vaghefi, Qahri-Saremi, and Turel [65]; and discontinuous usage intention from Avornyo et al. [85]. To ensure the validity of the questionnaire question items, we invited experienced users of short-video social media to conduct a pre-survey before the questionnaire was formally administered, then judging the presence of measurement items and ticked items that were “difficult to understand, vague, and with low differentiation”. Based on the survey results, some items were updated, added, deleted, and revised to generate a theoretical measurement scale of the influence mechanism of users’ discontinuous usage intention of pan-entertainment mobile live broadcast users.

### 4.2. Sample Characteristics

The introduction to the survey emphasized the importance of the participant’s contributions to the study and their role in advancing the field of pan-entertainment mobile live broadcast platform research. The participants were informed that their responses would be used solely for academic purposes and would not be shared with third parties. The anonymity and confidentiality of the study were also guaranteed, with no personal identifying information collected. A reward system was implemented to incentivize the participants further to provide honest and thoughtful responses. Each participant who completed the survey could receive a 10 RMB WeChat red packet. This reward was intended to thank the participants for their time and effort in completing the survey and to encourage them to answer each question truthfully and to the best of their ability. The use of WeChat red packets as a reward was chosen for their ease of use and accessibility to the participants. Overall, these measures were taken to ensure the data’s quality and accuracy and promote high participation rates. In this study, 425 questionnaires were collected from July to October 2022 through an online questionnaire platform (Questionnaire Star, a Chinese professional platform that provides service of online questionnaire survey), and 11 invalid questionnaires were excluded. The screening criteria include whether the respondents had experience in using pan-entertainment mobile live broadcast, and 8 questionnaires from respondents who had never used the platform were excluded; the second criterion is the respondents’ attitude in filling out the questionnaires, and the questionnaires with all or almost all the same answers were excluded, and the questionnaires with a response time of less than 15 s were excluded. A total of 15 questionnaires were excluded, and finally, 391 valid questionnaires were collected, with the effective rate of 92%. In terms of gender, 39.1% of the users are male and 60.9% are female. In terms of age, the largest number of users were aged 18–25, accounting for 56.3%; in terms of educational background, the largest number of users had a bachelor’s degree, accounting for 41.7%; and in terms of frequency of use, 42.2% of users used it frequently, as shown in Table 1. The distribution of basic information such as gender, age, and frequency of use of the questionnaire is consistent with the actual structure of Chinese pan-entertainment mobile live broadcast platform users, so it deserves further statistical analysis. The study was approved by the Academic Ethics Committee of X University in May 2022.

## 5. Results

### 5.1. Measurement Model Assessment

#### 5.1.1. Results of the Reliability and Validity Test

Internal consistency reliability is a statistical method used to measure the consistency and reliability of tests or actual measurements in the research. It determines the degree of measurement error, which ensures that the test results are stable, consistent, trustworthy, and reliable. Two commonly used methods for testing internal consistency reliability are Cronbach’s Alpha coefficient (CA) and Construct reliability (CR). This study used Cronbach’s α coefficient to measure the consistency of the questionnaire as a pre-test reliability measure. The α coefficient ranges from 0 to 1, with a value greater than 0.7 indicating that it is trustworthy and a value greater than 0.9 representing very high trustworthiness. The composite reliability value represents the internal consistency of the construct indicators, and a value of 0.7 is acceptable [86], with a recommended value of 0.6 or higher [87]. Factor loading analysis measures the correlation between individual variables and factors. The factor loading ranges from −1 to 1, and its square represents the percentage of variation that the factor can explain. In research, a threshold of 0.7 is commonly used as the standard, with values greater than this being acceptable [88]. Another method used in this study to test composite validity is the Average Variance Extracted (AVE), which represents the proportion of the observed variable values that the latent variable can measure. AVE is used to evaluate the reliability and discriminant validity and is considered good when its value exceeds 0.5 [88]. Overall, the reliability and validity analysis results for this study are presented in Table 2.

#### 5.1.2. Discriminant Validity

The discriminant validity analysis is to verify whether there is a statistical difference between two different constructs. The items in different constructs should not be highly correlated, and if they are (0.85 or higher), it means that these items are measuring the same thing, which often happens when the definitions of the constructs overlap excessively. In this study, a rigorous AVE method was used to evaluate the discriminant validity, and as per Fornell and Larcker [89], the square root of each factor’s AVE must be greater than the correlation coefficients of each pair of variables, indicating that the factors have discriminant validity. The diagonal is the square root of each factor’s AVE, greater than the standardized correlation coefficients outside the diagonal. This study has discriminant validity, and the lower triangle is the correlation coefficient. See the Table 3 below for details.

The heterotrait–monotrait ratio of validity, also known as the ratio of between-trait to within-trait correlations, is used to assess the discriminant validity of different constructs. It compares the average correlations between indicators of different constructs to the averages between indicators of the same construct. As shown in the Table 4, the results indicate that the HTMT values between each pair of variables in this study are below 0.85, indicating good discriminant validity for each variable.

### 5.2. Structural Equation Model

#### 5.2.1. Model Fit R^2^

In general, the R^2^ value of an endogenous latent variable that is greater than 0.67 indicates a strong explanatory power while values between 0.33 and 0.67 indicate moderate explanatory strength, values between 0.19 and 0.33 indicate low explanatory power, and values below 0.19 indicate virtually no explanatory power. The results of this study are shown in the table below, and it can be concluded that the explanatory R^2^ of Cognitive Dissonance is 0.486, indicating a moderate to strong explanatory percentage, and the explanatory R^2^ of Discontinuous Usage Intention is 0.432, also indicating a moderate to strong explanatory percentage, as shown in Table 5.

#### 5.2.2. Path Size Significance

**Figure 2:**
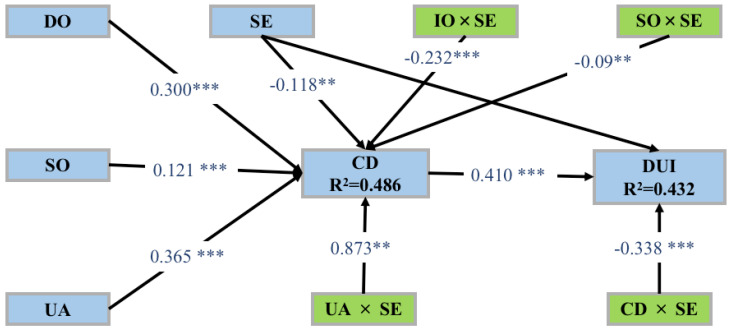
The effects of predictors of the discontinuous usage intention of pan-entertainment mobile live broadcast platforms. ** *p* < 0.01, *** *p* < 0.001.

The size and significance of path coefficients are used to evaluate the relationship between research hypotheses. After standardizing the sample data, path coefficients are between 1 and −1. A value closer to 1 indicates a stronger positive correlation, while a value closer to −1 indicates a stronger negative correlation. The T value can be further calculated by dividing the path coefficient by the standard deviation. According to previous studies, when the sample size is greater than 30, the critical value can be calculated using the normal distribution’s quartiles. Suppose the T value is greater than the critical value. In that case, it can be declared that it has a significant level within a certain error, and the critical value is usually 1.96 (5% significance level), 2.57 (1% significance level), and 3.29 (0.1% significance level). This study calculated the path coefficient and T value through bootstrapping with a sample of 5000 bootstrap cases. The results of the structural model’s path coefficients are shown in Table 6 and Figure 2.

The above Table 7 indicates that information overload has a significant positive effect on cognitive dissonance (β = 0.300, *p* < 0.001), service overload has a significant positive effect on cognitive dissonance (β = 0.121, *p* < 0.001), user addiction has a significant positive effect on cognitive dissonance (β = 0.365, *p* < 0.001), and cognitive dissonance has a significant positive effect on discontinuous usage intention (β = 0.410, *p* < 0.001), assuming hypotheses H1–H4 are supported.

#### 5.2.3. Moderating Effect

**Figure 3:**
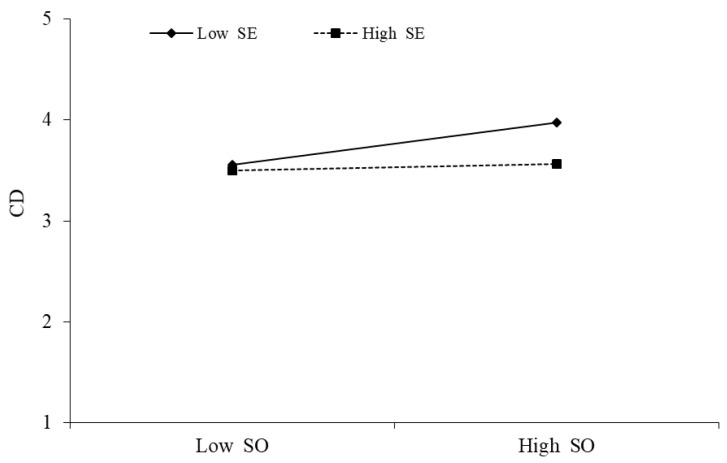
Moderating effect of SO × SE→CD.

The above Table 7 shows that User addiction × Self-efficacy has a significant negative effect on Cognitive dissonance (β = −0.120, *p* < 0.001), indicating the existence of moderation. That is, self-efficacy weakens the positive effect of user addiction on cognitive dissonance. This is supported by hypothesis H5, as shown in the following Figure 3.

**Figure 4:**
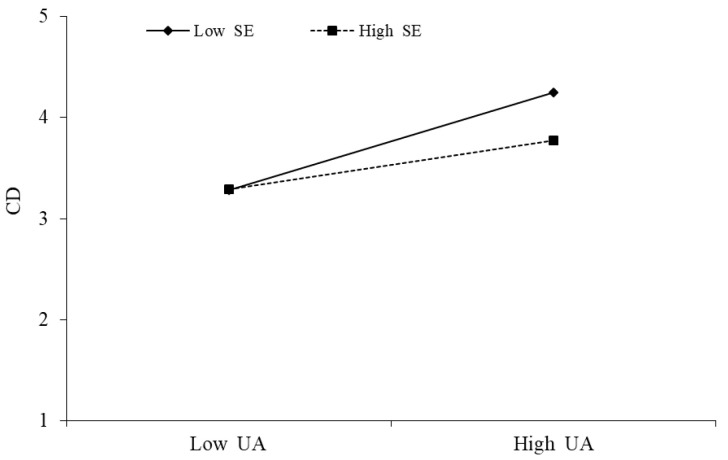
Moderating effect of UA × SE→CD.

The above Table 7 indicates that service overload × self-efficacy has a significant negative effect on cognitive dissonance (β = −0.090, *p* < 0.01), indicating the presence of moderation, meaning that self-efficacy reduces the positive impact of service overload on cognitive dissonance. This is supported by hypothesis H6, as illustrated in Figure 4.

**Figure 5:**
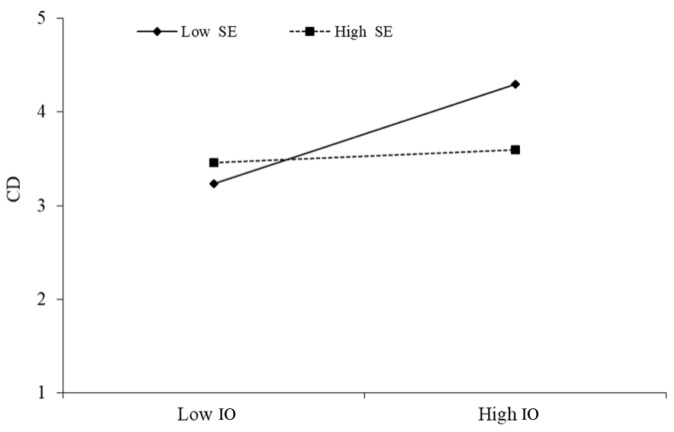
Moderating effect of IO × SE→CD.

As can be seen from the above Table 7, the interaction between information overload and self-efficacy has a significant negative impact on cognitive dissonance (β = −0.232, *p* < 0.001), indicating the presence of moderation. This means that self-efficacy weakens the positive impact of information overload on cognitive dissonance, assuming H7 is established, as shown in the following Figure 5.

**Figure 6:**
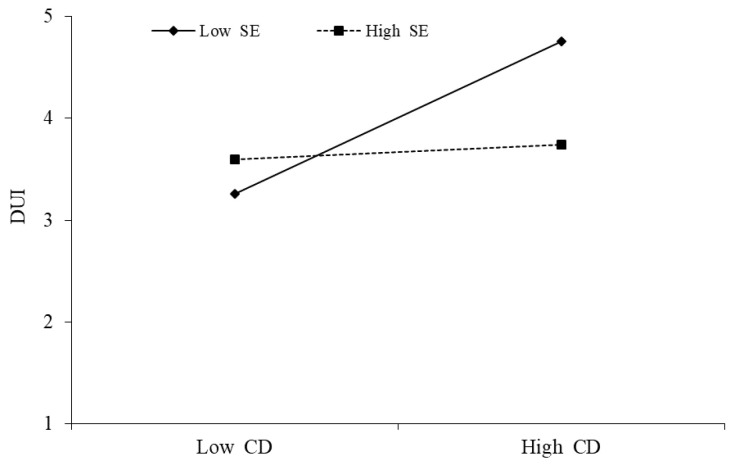
Moderating effect of CD × SE→DUI.

As can be seen from the above Table 7, the interaction between cognitive dissonance and self-efficacy has a significant negative impact on discontinuous usage intention (β = −0.338, *p* < 0.001), indicating the presence of moderation. This means that self-efficacy weakens the positive impact of cognitive dissonance on discontinuous usage intention, assuming H8 is established, as shown in Figure 6.

## 6. Discussion

### 6.1. Discussion of Key Findings

Based on the theoretical framework of “cognition-emotion-behavior intention”, this study constructs an influencing mechanism model of the discontinuous usage intention of pan-entertainment mobile live broadcast users from the perspective of cognitive dissonance and self-efficacy and conducts hypothesis testing and empirical analysis, then arrives at the following conclusions:

First, recent studies have highlighted the crucial role of the cognitive dimension in discontinuous usage intention. For example, Ye, Cho, Chen, and Jia [11] found a positive relationship between users’ perception of information overload and discontinuous usage intention in social media platforms. Moreover, the literature has widely documented the negative effects of user addiction on cognitive dissonance [90,91]. The findings of the present study are in line with these studies. Pan-entertainment mobile live broadcast users experience information overload, service overload, and user addiction, leading to cognitive dissonance and resulting in discontinuous usage intention. Homogeneous content domain overload and heterogeneous information impact contribute to cognitive dissonances, such as anxiety, guilt, and regret, while the platform’s functions may exceed individual cognitive and processing abilities. The individual heterogeneous cognitive structure and negative effects of user addiction can also trigger cognitive dissonance, which can be alleviated by the behavioral intention of stopping use or uninstalling it.

Second, a study found that users’ self-efficacy in managing information overload was negatively related to cognitive dissonance and positively related to continuous usage intention in social media platforms [65]. Moreover, Qaisar et al. [92] revealed that user addiction had a stronger negative effect on discontinuous usage intention among users with low self-efficacy towards mobile applications. Similarly, a recent study found that users with high self-efficacy had a more positive attitude towards discontinuing the use of social media platforms, whereas users with low self-efficacy were more likely to experience negative emotions and cognitive dissonance when discontinuing use [93]. Consistent with these findings, our study suggests that pan-entertainment mobile live broadcast users with high self-efficacy can better manage the effects of information overload, service overload, and user addiction on cognitive dissonance and are less likely to discontinue usage intention. In contrast, users with low self-efficacy are more susceptible to negative emotions and cognitive dissonance when using the platform, which can lead to discontinuous usage intention.

This study’s third finding highlights the relationship between cognitive and affective dimensions in users of pan-entertainment mobile live broadcast platforms. The results indicate that information overload, service overload, and user addiction positively impact users’ cognitive dissonance, which, in turn, leads to discontinuous usage intention. Additionally, the study found that self-efficacy negatively regulates the effect of cognitive dissonance and the impact of information overload, service overload, and user addiction on cognitive dissonance. Users with high self-efficacy are more likely to regulate the discomfort caused by cognitive dissonance and make behavioural decisions based on their cognitive and regulated emotional state. Conversely, users with low self-efficacy are more prone to discontinuous usage intention, potentially leading them to uninstall the platform. Recent literature supports the study’s findings on cognitive and affective dimensions in user behaviour. Zhang, Zhao, Lu, and Yang [79] research showed that information overload and service overload could lead to cognitive dissonance and negative emotions, resulting in discontinuous usage intention. Marikyan, Papagiannidis, and Alamanos [83] discovered a positive correlation between user addiction and cognitive dissonance, leading to discontinuous usage intention.

### 6.2. Theoretical Implications

The results of this study have several significant theoretical implications in the field of information systems and psychology. These implications are discussed below:

First, prior research in the field of information systems and psychology has demonstrated the importance of considering the interplay between cognitive, emotional, and behavioral factors in the study of user intentions, particularly in the context of technology adoption and usage. The “cognition-emotion-behavior intention” framework has been widely used to understand user intentions, but it has largely been limited to the consideration of cognitive factors, such as attitudes and beliefs [94]. This study extends the traditional “cognition-emotion-behavior intention” framework by incorporating the concepts of cognitive dissonance and self-efficacy in the study of discontinuous usage intentions of pan-entertainment mobile live broadcast platform users. Cognitive dissonance, defined as the psychological discomfort experienced by a person who holds two or more contradictory beliefs or values [95], has been found to be a significant predictor of user discontinuous usage intentions in prior research [43]. Self-efficacy, or the belief in one’s ability to perform a task effectively [45], has also been shown to be a key determinant of user behavior in the context of technology usage [45]. By incorporating both cognitive dissonance and self-efficacy in the “cognition-emotion-behavior intention” framework, this study provides a more comprehensive understanding of the discontinuous usage intentions of pan-entertainment mobile live broadcast platform users. The findings have practical implications for the design and development of pan-entertainment mobile live broadcast platforms, as they highlight the importance of considering both cognitive dissonance and self-efficacy in order to increase user satisfaction and reduce discontinuous usage intentions.

Second, cognitive dissonance in IS research is important to discuss. The concept of cognitive dissonance has been widely studied in the field of psychology for several decades [95]. However, its application in the field of information systems has been limited until recent years. Several studies have investigated the relationship between cognitive dissonance and different outcomes such as attitude change, behavior change, and consumer decision making [96,97]. This study adds to the prior research by providing empirical evidence for the role of cognitive dissonance in discontinuous usage intentions of pan-entertainment mobile live broadcast platform users. This contribution expands the application of the concept of cognitive dissonance in the field of information systems and offers new insights into the relationship between information overload, service overload, and user addiction and the role of cognitive dissonance in discontinuous usage intentions.

Third, the role of self-efficacy has been widely studied in the field of psychology, particularly in the areas of motivation, achievement, and mental health. Bandura [45] first introduced the concept of self-efficacy and defined it as “people’s beliefs about their capabilities to produce designated levels of performance that exercise control over events that affect their lives”. Self-efficacy has been found to play a critical role in determining an individual’s behavior, attitudes, and decisions, particularly in the face of challenges and stress [98]. In the context of information systems, self-efficacy has been studied in relation to various aspects of user behavior, such as technology adoption, usage behavior, and satisfaction [99]. The relationship between self-efficacy and information overload has been extensively studied in prior research. For example, Islam, Whelan, and Brooks [93] found that self-efficacy is negatively related to information overload. Similarly, Yener et al. [100] found that self-efficacy helps mitigate the impact of information overload on job stress and job burnout among information workers. This study extends prior research by exploring the role of self-efficacy in regulating the relationship between information overload, service overload, user addiction, and cognitive dissonance in the context of discontinuous usage intentions of pan-entertainment mobile live broadcast platform users. The findings suggest that self-efficacy plays a negative role in regulating the impact of cognitive dissonance on discontinuous usage intentions, which provides important insights for the design and development of pan-entertainment mobile live broadcast platforms.

Finally, information overload theory posits that when individuals are exposed to excessive information, their ability to process and comprehend the information is compromised, leading to cognitive overload and dissonance [101]. The study’s findings add to the body of literature supporting this theory, particularly in the context of pan-entertainment mobile live broadcast platforms. The study identifies information overload as a critical factor contributing to cognitive dissonance, leading to discontinuous usage intentions among platform users. This finding suggests that information overload may be a significant concern for platform operators, as it can result in negative user experiences and ultimately impact user retention. Therefore, platform operators should consider strategies to mitigate information overload, such as by providing filtering mechanisms or personalized recommendations to help users manage the amount of information they receive. Additionally, future research could explore how different types of information, such as user-generated content or platform-generated content, contribute to information overload and cognitive dissonance and how these factors can be managed to improve user experiences on social media platforms.

### 6.3. Implications for Practice

This study investigated the mechanism behind the discontinuous usage intention of users of pan-entertainment mobile live broadcasts. Utilizing the concept of cognitive dissonance and self-efficacy, it analyzed the interplay of cognition, affection, and behavioral intentions. The study also examined the impact of information overload, service overload, and user addiction on cognitive dissonance and revealed the mitigating effect of self-efficacy in these relationships. This study provides a new perspective on discontinuous usage in the context of social media and expands the application of the theories of cognitive dissonance and self-efficacy. The results of this study have practical implications for regulating user behavior and optimizing platform services.

First, in order to effectively meet the needs of users, platform designers should adopt a user-centered approach when optimizing the service function structure. This involves incorporating lightweight design principles to avoid service overload and cognitive dissonance, as well as enhancing the system functions, service quality, and user feedback channels of the pan-entertainment mobile live broadcast platform. In particular, a simplification of functions should be prioritized, including removing or altering uncommon functions while emphasizing frequently used features, such as the ability to pause or return in the live broadcast interface. Additionally, it is crucial to optimize the system and improve responsiveness to reduce internet lag, which has a negative impact on user experience. Continuous innovation should also be emphasized, as it is a driving force for technological development and can contribute to the generation of new functional experiences, thereby attracting more customers.

In addition to optimizing the service function structure and system functions, it is important for the platform operators to consider the demographic characteristics of users when enhancing the user experience of the pan-entertainment mobile live broadcast platform. This involves taking a balanced approach to the age, gender, and geographical distribution of users, and vertically segmenting content to meet the needs of different user groups. To reduce cognitive dissonance caused by information overload and service overload, personalized information reception and interaction services should be provided, taking into account the core needs of the platform’s users. To reduce cognitive dissonance, there are three effective strategies: changing user behavior, modifying perceptions, and introducing new perceptions. By empowering users with control over their experience on the platform, such as the ability to customize their live feed or control the amount of content they are exposed to, the platform can help alleviate information overload and increase user agency. Additionally, educational measures, such as providing guidance on how to effectively filter content or use platform features, can also play a role in reducing cognitive dissonance and improving the overall user experience. The study suggests that self-efficacy can play a key role in moderating the impact of the cognitive dissonance on discontinuous usage intentions. Platforms should consider implementing moderation features that help users manage their self-efficacy and reduce cognitive dissonance, encouraging more consistent usage.

To drive innovation and enhance the user experience, platform development engineers should leverage cutting-edge technologies, such as metaverse, cloud computing, and blockchain in the development of the pan-entertainment mobile live broadcast platform. This can optimize the live channel recommendation algorithm and personalize the platform to individual users. Furthermore, AI can be utilized to strengthen network security and privacy protection, ensuring the secure storage and protection of user data. One approach that has gained popularity for enhancing privacy protection is Federated Learning [102]. This technology employs a distributed machine learning approach, which assumes that users’ data is not stored on a centralized server but is instead kept confidential on the users’ edge devices, such as smartphones. By training the AI model on the user’s mobile device and transferring only information about the parameters obtained from the training back to a global model, Federated Learning can significantly improve user privacy compared to traditional machine learning approaches. While data collection is a necessary aspect of AI-driven systems, it is equally important to implement technical measures to prevent unauthorized disclosure of personal information, thereby preserving the privacy of users.

Lastly, platform supervisors should enhance regulation and management, establishing user hierarchy management, implementing and standardizing content review systems, and constructing an advanced content classification and identification system. Meanwhile, the interaction between anchors and users in the pan-entertainment mobile live broadcast platform should be improved to shape the users’ self-efficacy and mitigate the adverse effects of information overload, service overload, and emotional cognitive dissonance brought on by addiction. In addition, users should also foster media literacy, meaning they should assess their own needs and benefits from using media rationally, avoid a passive dependence on the media, and make the pan-entertainment mobile live broadcast platform a commercially beneficial product. Whether it is through platform or government regulation, users ultimately play an important role, and if users can consciously improve their own qualities, user management will be more effective with less effort. Prior studies have also shown that cyberbullying is a major concern among pan-entertainment mobile live broadcast platform users [103]. To tackle this issue, platforms should implement strict policies and procedures for handling cyberbullying and provide users with the ability to report and block other users.

## 7. Research Limitations and Future Research Directions

This study has several limitations that should be considered when interpreting the results. First, other potential factors contributing to discontinuous usage intentions among pan-entertainment mobile live broadcast platform users were not examined. These factors could include the quality of content, the reliability of the platform, and the level of user engagement. Future research could investigate these factors to gain a more comprehensive understanding of discontinuous usage intentions. Second, the study used a cross-sectional survey design with a single source of data, which limits the ability to establish causality and the changes in discontinuous usage intentions over time. A longitudinal research design with multiple data sources could provide more insights into these issues. Third, the study focused on the Chinese market, and the results may not be generalizable to other countries and cultures. Future research could examine users’ discontinuous usage intentions in different contexts to compare and contrast the results. Finally, due to the interference of endogeneity issues, we cannot make further causal inferences and may think further about the issue if future survey data allow.

## 8. Conclusions

The study aimed to explore the discontinuous usage intentions of pan-entertainment mobile live broadcast users and the role of cognitive dissonance and self-efficacy in this process. The findings of the study extend the traditional “cognition-emotion-behavior intention” framework by incorporating the concepts of cognitive dissonance and self-efficacy and provide a more comprehensive understanding of discontinuous usage intentions. The results of the study offer empirical evidence for the role of cognitive dissonance in discontinuous usage intentions and provide insights into the relationship between information overload, service overload, user addiction, and cognitive dissonance. Moreover, the study highlights the significance of self-efficacy in regulating the relationship between information overload, service overload, user addiction, and cognitive dissonance. The results show that self-efficacy plays a negative role in regulating the impact of cognitive dissonance on discontinuous usage intentions, which is important for the design and development of pan-entertainment mobile live broadcast platforms.

## Figures and Tables

**Table 1:** Descriptive statistics of sociodemographic variables (N = 391).

Measure	Category	Frequency	Percentage (%)
Gender	Male	153	39.130
	Female	238	60.870
Age	<18	11	2.813
	18-25	209	53.453
	26-35	149	38.107
	36-45	14	3.581
	>45	8	2.046
Education	High school or below	22	5.627
	Junior college	24	6.138
	Bachelor	163	41.688
	Master	155	39.642
	Doctor	27	6.905
Frequency of using pan-entertainment mobile live broadcast platforms	Rarely use	131	33.504
	Occasionally use	95	24.297
	Frequently use	165	42.199

**Table 2:** Reliability and validity analysis.

Measurements	Path	Factor Loading	Standard Error	T	*p*	CA	CR	AVE
Cognitive dissonance (CD)	CD1←CD	0.853	0.015	56.303	0.000	0.831	0.899	0.747
	CD2←CD	0.873	0.012	74.614	0.000			
	CD3←CD	0.867	0.012	72.382	0.000			
Information overload (IO)	IO1←IO	0.830	0.017	47.783	0.000	0.866	0.908	0.712
	IO2←IO	0.870	0.013	65.725	0.000			
	IO3←IO	0.834	0.018	47.032	0.000			
	IO4←IO	0.842	0.017	50.087	0.000			
Discontinuous usage intention (DUI)	DUI1←DUI	0.843	0.015	54.983	0.000	0.808	0.886	0.723
	DUI2←DUI	0.879	0.010	87.304	0.000			
	DUI3←DUI	0.828	0.020	42.138	0.000			
Self-efficacy (SE)	SE1←SE	0.829	0.019	44.130	0.000	0.849	0.898	0.687
	SE2←SE	0.848	0.019	45.427	0.000			
	SE3←SE	0.828	0.019	44.196	0.000			
	SE4←SE	0.811	0.021	38.151	0.000			
Service overload (SO)	SO1←SO	0.885	0.016	55.839	0.000	0.857	0.913	0.777
	SO2←SO	0.901	0.013	69.136	0.000			
	SO3←SO	0.859	0.025	33.814	0.000			
User addiction (UA)	UA1←UA	0.848	0.014	59.344	0.000	0.855	0.901	0.696
	UA2←UA	0.828	0.018	46.525	0.000			
	UA3←UA	0.844	0.016	52.974	0.000			
	UA4←UA	0.816	0.018	44.344	0.000			

**Table 3:** Discriminant validity.

	IO	SO	UA	SE	CD	DUI
IO	0.844					
SO	0.189	0.882				
UA	0.264	0.219	0.834			
SE	−0.288	−0.218	−0.233	0.829		
CD	0.429	0.280	0.516	−0.318	0.864	
DUI	0.459	0.321	0.432	−0.340	0.542	0.850

**Table 4:** HTMT discriminant validity.

	IO	SO	UA	SE	CD	DUI
IO						
SO	0.219					
UA	0.297	0.25				
SE	0.333	0.253	0.269			
CD	0.497	0.332	0.607	0.374		
DUI	0.544	0.383	0.516	0.403	0.657	

**Table 5:** Model fit R^2^.

	R^2^	Adjusted R^2^
CD	0.486	0.476
DUI	0.432	0.428

**Table 6:** Path Coefficients of the PLS Structural Equation Model.

Path	Path Coefficient	Standard Deviation	T	*p*	95% Upper Interval	95% Lower Interval
IO→CD	0.300	0.041	7.235	0.000	0.217	0.380
SO→CD	0.121	0.037	3.308	0.001	0.046	0.192
UA→CD	0.365	0.039	9.408	0.000	0.285	0.439
SE→CD	−0.118	0.042	2.849	0.004	−0.197	−0.034
SE→DUI	−0.167	0.045	3.751	0.000	−0.253	−0.080
CD→DUI	0.410	0.043	9.612	0.000	0.321	0.487
IO × SE→CD	−0.232	0.037	6.270	0.000	−0.305	−0.162
SO × SE→CD	−0.090	0.034	2.641	0.008	−0.159	−0.025
UA × SE→CD	−0.120	0.039	3.063	0.002	−0.197	−0.044
CD × SE→DUI	−0.338	0.039	8.588	0.000	−0.417	−0.260

**Table 7:** Moderating effect.

Path	Path Coefficient	Standard Deviation	T	*p*	95% Upper Interval	95% Lower Interval
IO × SE→CD	−0.232	0.037	6.270	0.000	−0.305	−0.162
SO × SE→CD	−0.090	0.034	2.641	0.008	−0.159	−0.025
UA × SE→CD	−0.120	0.039	3.063	0.002	−0.197	−0.044
CD × SE→DUI	−0.338	0.039	8.588	0.000	−0.417	−0.260

## Data Availability

Not applicable.
